# Preferential loss of a polymorphic *RIZ* allele in human hepatocellular carcinoma

**DOI:** 10.1054/bjoc.2000.1667

**Published:** 2001-03

**Authors:** W Fang, Z Piao, I M Buyse, D Simon, J C Sheu, M Perucho, S Huang

**Affiliations:** Program in Oncogenes and Tumor Suppressor Genes, The Burnham Institute, La Jolla, CA, 92037; Department of Pathology, MCP-Hahnemann School of Medicine, Philadelphia, PA, 19109; Department of Internal Medicine, National Taiwan University Hospital, No. 1 Chang-Te street, Taipei, Taiwan

**Keywords:** *RIZ* (PRDM2), polymorphisms, hepatoma

## Abstract

The *RIZ (PRDM2)* locus commonly undergoes loss of heterozygosity (LOH) and maps within the minimal deleted region on 1p36 in hepatocellular carcinoma (HCC). Although peptide-altering mutations of *RIZ* are rare in HCC, the RIZ1 product is commonly lost in HCC and has tumour suppressive activities. Here, we analysed *RIZ* gene mutations and LOH in HCC, breast cancer, familial melanoma, colon cancer, and stomach cancer. We found 7 polymorphisms but no mutations. By analysing the Pro704-deletion polymorphism, we detected LOH of *RIZ* in 31 of 79 (39%) informative HCC cases, 11 of 47 (23%) colon cancer cases, 8 of 43 (19%) breast cancer cases, 8 of 66 (12%) stomach cancer cases. Importantly, loss of the Pro704^+^allele was found in 74% of the 31 LOH positive HCC cases (*P*< 0.01), indicating a preferential loss and hence a stronger tumour suppressor role for this allele compared to the P704^−^allele. In addition, the Pro704^+^allele was found to be more common in Asians (0.61) than Caucasians (0.42) (*P* = 0.0000), suggesting an interesting link between gene polymorphisms and potential differences in tumour incidence between racial groups. © 2001 Cancer Research Campaign http://www.bjcancer.com


British Journal of Cancer (2001) **84**(6), 743–747
© 2001 Cancer Research Campaign
doi: 10.1054/ bjoc.2000.1667, available online at http://www.idealibrary.com on IDEAL®

http://www.bjcancer.com

### Preferential loss of a polymorphic *RIZ* allele in human hepatocellular carcinoma

**W Fang[1], Z Piao[1], IM Buyse[1#], D Simon[2], JC Sheu[3], M Perucho[1] and S Huang[1]**

[1]Program in Oncogenes and Tumor Suppressor Genes, The Burnham Institute, La Jolla, CA 92037; [2]Department of Pathology, MCP-Hahnemann School of Medicine, Philadelphia, PA 19109; [3]Department of Internal Medicine, National Taiwan University Hospital, No. 1, Chang-Te street, Taipei, Taiwan

**Summary** The *RIZ (PRDM2)* locus commonly undergoes loss of heterozygosity (LOH) and maps within the minimal deleted region on 1p36 in hepatocellular carcinoma (HCC). Although peptide-altering mutations of *RIZ* are rare in HCC, the RIZ1 product is commonly lost in HCC and has tumour suppressive activities. Here, we analysed *RIZ* gene mutations and LOH in HCC, breast cancer, familial melanoma, colon cancer, and stomach cancer. We found 7 polymorphisms but no mutations. By analysing the Pro704-deletion polymorphism, we detected LOH of *RIZ* in 31 of 79 (39%) informative HCC cases, 11 of 47 (23%) colon cancer cases, 8 of 43 (19%) breast cancer cases, 8 of 66 (12%) stomach cancer cases. Importantly, loss of the $Pro704^+$ allele was found in 74% of the 31 LOH positive HCC cases ($P < 0.01$), indicating a preferential loss and hence a stronger tumour suppressor role for this allele compared to the $P704^-$ allele. In addition, the $Pro704^+$ allele was found to be more common in Asians (0.61) than Caucasians (0.42) ($P = 0.0000$), suggesting an interesting link between gene polymorphisms and potential differences in tumour incidence between racial groups. © 2001 Cancer Research Campaign http://www.bjcancer.com

**Keywords**: *RIZ* (PRDM2); polymorphisms; hepatoma

Inactivation of tumour suppressor genes plays an important role in human cancer formation and progression. The distal short arm of chromosome 1 or 1p36 is thought to harbour several tumour suppressor genes because loss of heterozygosity (LOH) in this region is common in a large number of different types of human cancers (Weith et al, 1996). One candidate in this region is the retinoblastoma protein-interacting zinc finger gene *RIZ(PRDM2)*, which was isolated in a functional screening for Rb-binding proteins (Buyse et al, 1995), also independently isolated as a DNA-binding protein MTB-Zf (Muraosa et al, 1996), a GATA3 transcription factor binding protein G3B (Shapiro et al, 1995), and a coactivator of oestrogen receptor (ER) (Abbondanza et al, 2000). The gene maps within the minimal deleted region on 1p36 in liver, breast and familial colon cancers (Chadwick et al, 2000; Fang et al, 2000).

RIZ is a member of a gene family which shares a ~ 130 amino acid motif called the PR (*P*RDI-BF1 and *R*IZ) domain, also termed SET (*S*uvar3-9, *E*nhancer-of-zeste, *T*rithorax); the family is known to play an important role in chromatin-mediated gene expression, development and cancer (Huang et al, 1998). The PR/SET domain shows significant homology with plant protein lysine methyltransferase (MTase); the PR/SET domain of SUV39H1 can methylate histone H3 and plays an important role in chromatin condensation during mitosis (Rea et al, 2000). The PR/SET domain represents the catalytic core motif and appears to define a large family of protein lysine methyltransferases.

The *RIZ* gene produces 2 products through alternative promoters, RIZ1 that contains the PR domain and RIZ2 lacking the motif (Liu et al, 1997). Decreased or lost expression of RIZ1 mRNA but not of RIZ2 is found in all human cancers examined, including those of breast, liver, lung, colon, and neuroendocrine tissues, suggesting a tumour suppressor role for the RIZ1 product (He et al, 1998; Jiang et al, 1999; Chadwick et al, 2000). In addition, frequent frame shift mutations of *RIZ* appear selected in gastrointestinal, endometrial, and pancreatic tumours associated with microsatellite instability (Chadwick et al, 2000; Piao et al, 2000; Sakurada et al, 2001). Together, these findings show frequent inactivation of RIZ1 in a broad spectrum of human cancers. Consistently, recombinant adenovirus-mediated RIZ1 expression can induce G2/M cell cycle arrest, apoptosis, or both in several tumour cell lines (He et al, 1998; Jiang et al, 1999; Chadwick et al, 2000).

To further determine the role of *RIZ* in HCC and other 1p36-linked human cancers, we here studied *RIZ* gene mutations and LOH in HCC, breast cancer, familial melanoma, colon cancer and stomach cancer. The previously described Pro704-deletion polymorphism (Fang et al, 2000) was analysed for its potential differential involvement in carcinogenesis. The distribution of this polymorphism in Asian and Caucasian populations were also determined.

### MATERIALS AND METHODS

### Tissue DNAs

Blood DNAs of 6 patients from 6 different melanoma families were examined for peptide-altering mutations in the entire coding region of RIZ1 by SSCP analysis. The identification numbers of these DNAs were 7559206, 3234202, 0720200, 5864201, 0617200, and 0099204. These DNAs have been described previously (Bale et al, 1989).

147 HCC cases were included in the study. Among these, 39 were collected at the Yonsei University Medical Center, Seoul,

*Received 21 June 2000*
*Revised 20 November 2000*
*Accepted 29 November 2000*

*Correspondence to:* S Huang

[#]Present address: Department of Molecular and Human Genetics, Baylor DNA Diagnostic Laboratory, Baylor College of Medicine, One Baylor Plaza, Houston, TX77030.

Korea, which have been described previously (Piao et al, 1998a, 1998b); 10 were collected at the University of Miami and University of Pittsburgh, which have been described in Hammond et al (1999) and (Simon et al 1991); 98 were collected at the National Taiwan University Hospital, which have been described in (Lin et al, 1999).

40 breast cancer cases, 46 primary colon cancer cases, and 86 stomach cancer cases were also included in this study which were collected at the Yonsei University Medical Center, Seoul, Korea. In addition, 60 colon cancer cases, 39 stomach cancer cases, and 41 breast cancer cases were obtained from the Connective Tissue Network at the University of Alabama; the DNA from these tumours and their adjacent non-tumoral tissues were extracted using standard procedures.

DNAs from 30 normal Caucasian individuals were collected at the University of Leuven, Belgium. In addition, DNAs from 13 normal Caucasian individuals were collected at McGill University, Montreal, Quebec (kind gift of Dr C Polychronakos of McGill University).

### SSCP, allelotyping, and DNA sequencing analysis

PCR primers for SSCP analysis of the PR domain region of RIZ1 including exons 2–7 were described previously. PCR primers for the remaining part of RIZ1 gene including exons 8–9 are listed in Table 1. PCR reactions were carried out in a mixture of 20 µl containing 1.5 mM $MgCl_2$, 20 pmol primer, 0.2 mM each dATP, dGTP, dTTP, 5 µM dCTP, 1 µCi α-$^{32}$P-dCTP (3000 Ci $mmol^{-1}$; NEN DuPont, Boston, MA), 25 ng of sample DNA, 1X PCR buffer and 1.25 U Taq polymerase. After denaturation at 95˚C for 4 min, DNA amplification was performed in 25–30 cycles consisting of denaturation at 95˚C for 30 s, primer annealing at

**Table 1** PCR primers for SSCP analysis of *RIZ* gene

| Primers | Coordinates[a] | Sequences (5′ to 3′) |
|---|---|---|
| RP111[b] | intron 8 (622) | CTT CTG CTT CCA TGT GCT |
| RP112 | 888 | CCC TCG TCT TCC AAC TC |
| RP110[c] | 891 | AGA AGA AGC CAG CAT GCC |
| RP142 | 1455 | GCA CGG ATG AAG TTC TTT AA |
| RP143[c] | 1413 | GTC GTA GAA GAG AAT GGG |
| RP118 | 2008 | CTC ACT ATT TGT GCT GCC |
| RP144 | 1888 | GGC AGC ACA AAT AGT GAG |
| RP105 | 2358 | ACT CCA TGC TGG TGA GTC |
| RP29[c] | 2349 | AGC ATG GAG TTT GTC TG |
| RP124 | 2828 | GGT GTG GAC TCA ACA GT |
| RP88 | 2771 | AGA TCC TGA CCT CGG TC |
| RP125 | 3056 | GAC TGT GCG GTG GCA T |
| RP90 | 2998 | CTT CCA GTG CAT CTC CAC A |
| RP92 | 3341 | CTG GGT TTC AGA CCT TCA |
| RP91 | 3357 | TGC TGC TGC ACA GGA TGT |
| RP126 | 3635 | CGC TGG TGC TGC TGC A |
| RP127 | 3569 | ATC TTT GTG TGT TCT GT |
| RP51 | 3817 | TCG TGT AAA GCT CTT CAG |
| RP128 | 3780 | GAG GAG TTA AAT GAT TC |
| RP140 | 4089 | CTT CTT GTC ACT TGC AGA |
| RP129 | 4024 | GGT GTC GAC AAT ATG CC |
| RP130 | 4298 | GCT TTC TGT ACT AGC TG |
| RP132 | 4237 | TGT CGT CGA ATA AGC TC |
| RP146 | 4499 | TGT CCA CCT TTC TTA GA |
| RP134 | 4423 | GCC GCC TTC AGC TGT CC |
| RP107 | 4670 | TTC TGC TTG GAC CTG AAG |
| RP135 | 4593 | TTG GGC AAG ACC AGA GC |
| RP147 | 4812 | AAG CTG AGC AGA ATG A |
| RP60 | 4734 | CCG ATA AGA ATG GCC AAA |
| RP137 | intron (5039) | CCC ACC AGC TCC TGA GC |
| RP95 | 5039 | CTA CAG CCT CCG CTT GGC G |
| RP100 | 5174 | AGC AGC CAG AGT GTC TA |

[a]The first nucleotide of the human RIZ1 cDNA coding region is designated 1. [b]These primers are located within introns; the positions of the exon boundaries are listed in brackets. [c]The PCR product of RP110+RP142 was digested with NsiI before SSCP gel analysis. The PCR product of RP143+RP118 was digested with HapII before SSCP gel analysis. The PCR product of RP29+RP134 was digested with EcoRI before SSCP gel analysis.

 © 2001 Cancer Research Campaign

55–60˚C for 30 s, and elongation at 72˚C for 30 s. GC-melt kit (Clontech, CA) was used to PCR the GC rich exon 6. Amplified DNA was diluted 2-fold with stop solution (95% formamide, 20 mM EDTA, 0.05% xylene cyanol and 0.05% bromophenol blue). 3 microlitres of amplified product were loaded onto MDE gel (FMC BioProducts, Rockland, ME 04841) for SSCP analysis or 6% polyacrylamide gel containing 5.6 M urea. For some PCR products, restriction digestion was performed before loading. The gel was dried on filter paper and exposed to Kodak XAR-5 film. Allelic loss was scored when band intensity of one allelic marker was significantly decreased (more than 70% reduction) in tumour DNA as compared with that in normal DNA. All variants detected by SSCP analysis were confirmed by DNA sequencing analysis.

### Statistical analysis

We correlate the LOH frequency with several clinical aspects, including sex, age, tumour size, and status of hepatitis B or C virus infection. The statistical analysis was performed by the computer program STATISTICA (StatSoft, Tulsa, OK, USA). The $P$ values were obtained by Chi-square test.

### RESULTS

### Lack of peptide altering mutations in RIZ1 in familial melanoma and breast cancers

A locus for familial cutaneous malignant melanoma-dysplastic naevus has been mapped to the region between an anonymous DNA marker (D1S47) and the gene locus for pronatrodilatin (NPPA) (Bale et al, 1989). To determine whether familial malanoma may carry peptide altering mutations in *RIZ* which maps just distal to NPPA, we performed SSCP analysis of DNAs from 6 familial melanoma patients. We did not detect mutations in the entire coding region of RIZ1 but found several polymorphic alleles of *RIZ* as shown in Table 2. 4 of these were silent nucleotide substitutions without affecting amino acid sequences. 2 were amino-acid substitutions, D283E and S450N. One was a deletion of a proline at codon 704 that has previously been described (Fang et al, 2000). We also scanned the PR domain region of RIZ1 in 61 breast cancer cases by SSCP analysis. We did not find any mutations besides the polymorphisms.

### LOH of RIZ in human cancers and preferential deletion of the Pro704$^+$ allele in HCC

Because of the relatively high rate of heterozygosity of the Pro704-deletion polymorphism (~50%), we used it to study whether LOH at the *RIZ* locus may be common in human cancers. We were also interested to determine whether this polymorphism may show differential linkage with human cancers. A total of 459 tumour cases were analysed and 235 of these were heterozygous for Pro704-deletion polymorphism as shown in Table 3. The tumours studied included HCC, breast cancer, colon cancer and stomach cancer. 58 of the 235 (25%) informative cases showed LOH at the *RIZ* locus. LOH was most common in HCC (31 of 79 or 39%), followed by colon carcinoma (11 of 47 or 23%), breast cancer (8 of 43 or 19%), and stomach cancer (8 of 66 or 12%). LOH of *RIZ* appeared more common in metastatic colon cancer (3/7) than primary colon carcinoma 8/40, but there was no statistical significance. No significant association of *RIZ* LOH with any clinical parameters of these tumours was observed,

Notably, the Pro704$^+$ allele was lost in 74% of the 31 LOH-positive HCC cases, indicating a significant preferential loss of this allele compared to the Pro704$^-$ allele ($P$ < 0.01) (Figure 1). This preferential loss of Pro704$^+$ allele did not correlate with any clinical parameters of HCC, including gender, age, tumour size, serum α-fetoprotein, and status of hepatitis B or C virus infection. A larger sample size will be needed to determine whether preferential loss of Pro704$^+$ allele was also significant in breast cancer

**Table 2** *RIZ* polymorphic variants

| Variant forms | PCR primers | Het frequency |
|---|---|---|
| R100 (CGA) to R100 (CGG) | RP257<br>RP258 | ND[a] |
| D283 (GAT) to E283 (GAA) | RP111<br>RP112 | 35.7% ($n$ = 28) |
| H364 (CAT) to H364 (CAC) | RP110<br>RP142 | 37.5% ($n$ = 8) |
| S450 (AGT) to N450 (AAT) | RP110<br>RP142 | 12.5% ($n$ = 8) |
| P704 (CCT) deletion | RP145<br>RP105 | 50.8% ($n$ = 502) |
| S1609 (TCG) to S1609 (TCA) | RP60<br>RP137 | 37.5% ($n$ = 8) |
| P1707 (CCG) to P1707 (CCA) | RP95<br>RP100 | N.D. |

[a]ND = not determined.

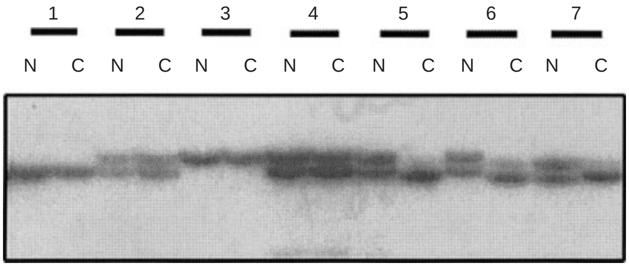

**Fig. 1** Preferential loss of P704$^+$ allele in human HCC. Representative autoradiographs of LOH analysis of the Pro704-deletion polymorphism. The HCC (C) and matched non-tumour tissue (N) are shown with case numbers indicated on the top. Tumour cases 5–7 showed loss of the upper band representing the P704$^+$ allele.

**Table 3** LOH of *RIZ* locus in human cancers

| | P704$^+$ del. | P704$^-$ del. | LOH rate |
|---|---|---|---|
| HCC | 23 | 8 ($P$ < 0.01) | 31/79 (39%) |
| Colon cancer | 6 | 5 | 11/47 (23%) |
| Breast cancer | 5 | 3 | 8/43 (19%) |
| Stomach cancer | 5 | 3 | 8/66 (12%) |
| Total | 39 | 19 | 58/235 (25%) |

**Table 4** Distribution of the Pro704-deletion polymorphism in Korean HCC and non-HCC patients

| | P704$^+$ hom | P704$^-$ hom | Het | Total | P704$^-$ freq |
|---|---|---|---|---|---|
| HCC[a] | 11 | 7 | 21 | 39 | 0.449 |
| non-HCC[a] | 64 | 21 | 87 | 172 | 0.375 |

[a]Genomic DNAs of normal tissues from cancer patients were used for the study.

*© 2001 Cancer Research Campaign* 

(63% of 8 LOH positive cases), stomach cancer (63% of 8 LOH positive cases), and colon cancer (55% of 11 LOH positive cases).

Given the differential involvement of the Pro704-deletion polymorphism with HCC, we next asked whether the Pro704$^-$ allele may be more enriched in HCC patients relative to non-HCC patients. Because different racial groups showed different allele frequency (see below), we limited our analysis to Korean patients. The Pro704$^-$ allele frequency in Korean HCC patients (0.449) was higher than that in non-HCC patients (0.375) (Table 4). But, this was not statistically significant ($P = 0.23$).

### Distribution of the Pro704-deletion polymorphism in Caucasian and Asian populations

Analysis of DNAs from non-tumour tissues of 150 Caucasian cancer patients from America showed that Pro704$^+$ allele (0.43) was less common than the Pro704$^-$ allele (Table 5). This allele frequency was found to be similar among different groups of patients with different cancers, indicating no preferential association of this allele with any specific type of cancers. 43 normal Caucasians from Canada and Belgium were also analysed and the Pro704$^+$ allele (0.36) was again less common. The results did not show significant variation between Caucasian normal and patient populations in the distribution of the Pro704-deletion polymorphism.

The distribution of the Pro704-deletion polymorphism was studied in 309 Asian cancer patients using their normal tissue DNAs. The Pro704$^+$ allele was the more common allele (0.61). This is significantly different from Caucasian cancer patient or normal populations ($P = 0.0000$).

### DISCUSSION

The RIZ1 product of the *RIZ* locus on 1p36 has been suggested as a candidate tumour suppressor. Although it maps within the region thought to harbour the familial melanoma locus, our data here suggest that peptide-altering mutations in *RIZ* may not be common in familial melanoma. Whether other types of *RIZ* alterations may be involved in this disease remains to be investigated.

We showed that LOH of *RIZ* was common but that peptide-altering mutations in RIZ1 were rare in breast cancers. The apparent selection for RIZ2 expression in breast cancers may have favoured the strategy of RIZ1 gene silencing rather than altering RIZ1 peptide sequences. This is similar to previous findings of lack of RIZ1 mutations in HCC where RIZ2 expression is uniformly present (Jiang et al, 1999; Fang et al, 2000). These observations suggest that decreasing RIZ1 expression may represent the more common way of inactivating RIZ1, at least in breast cancer and HCC. Recent data suggest that decreased RIZ1 expression was through DNA methylation of promoter CpG island (Y. Du and S.H., in preparation). While the importance of genetic mutations in cancer has long been recognized, the appreciation of epigenetic inactivation is more recent (Jones and Laird, 1999; Baylin and Herman, 2000; Eng et al, 2000). Recent studies have firmly established methylation as one potential hit in a modified Knudson's two-hit model (Jones and Laird, 1999). Thus, RIZ1 gene silencing and LOH could together achieve the two-hit inactivation of RIZ1.

Human HCC, colon carcinoma, and breast cancers are known to show LOH of 1p36 markers (Genuardi et al, 1989; Simon et al, 1991; Bardi et al, 1993; Kuroki et al, 1995). The minimal deleted regions in these cancers include the *RIZ* locus. Our results here confirm previous findings and directly demonstrate LOH of *RIZ* in these cancers. To the best of our knowledge, there has been no published report of LOH of 1p36 in stomach cancers. Here, we detected low frequency of LOH of *RIZ* in stomach cancer (12% in 66 stomach cancer cases), which indicates either a minor role of *RIZ* or 1p36 in this cancer or simply background rate chromosomal instabilities.

The preferential loss of the Pro704$^+$ allele in HCC suggests that this allele may be a stronger suppressor allele than the Pro704$^-$ allele. This notion would predict that individuals homozygous for the Pro704$^-$ allele would be more susceptible to tumour formation than those who are homozygous or heterozygous for the Pro704$^+$ allele. At least two observations are consistent with this prediction. First, our data did indicate a trend toward enrichment of the Pro704$^-$ allele in Korean HCC patients (Table 4).

**Table 5** Distribution of the Pro704-deletion polymorphism

| | P704$^+$ hom | P704$^-$ hom | Het | Total |
|---|---|---|---|---|
| Caucasian patients (P704$^+$ frequency = 0.43)[a] | | | | |
| US HCC | 3 | 2 | 5 | 10 |
| US colon cancer | 12 | 22 | 26 | 60 |
| US breast cancer | 3 | 16 | 22 | 41 |
| US stomach cancer | 9 | 9 | 21 | 39 |
| Total | 27 (17.5%) | 49 (32.5%) | 74 (50%) | 150 |
| Caucasian normal (P704$^+$ frequency = 0.37) | | | | |
| Canadian | 1 | 6 | 6 | 13 |
| Belgian | 5 | 12 | 13 | 30 |
| Total | 6 (14%) | 18 (41.9) | 19 (44.2%) | 43 |
| Asian patients (P704$^+$ frequency = 0.61)[a] | | | | |
| C. HCC[b] | 34 | 11 | 53 | 98 |
| K. HCC | 11 | 7 | 21 | 39 |
| K. breast cancer | 16 | 3 | 21 | 40 |
| K. colon cancer | 14 | 11 | 21 | 46 |
| K. stomach cancer | 34 | 7 | 45 | 86 |
| Total | 109 (34.9%) | 39 (13.1%) | 161 (51.9%) | 309 |

[a]Genomic DNAs of normal tissues from cancer patients were used for the study. [b]C = Chinese; K = Korean.

*© 2001 Cancer Research Campaign*

Second, the Pro704$^-$ allele is more enriched in Caucasian population compared to Asian population, which correlates with the known higher tumour incidence of Caucasians (see below). Nonetheless, this prediction needs to be further verified by future carefully controlled epidemiological studies. Also, the role of the Pro704 deletion polymorphism in non-HCC cancers will need to be further determined.

Our data show that the Pro704$^+$ allele of *RIZ* is significantly more common in cancer patients of Korean and Chinese than in those of Caucasian. Because we did not observe significant difference between cancer patients and normal individuals of Caucasian origin in the distribution of the Pro704$^+$ allele, we conclude that the allele frequency observed in Asian cancer patients is largely representative of normal populations. In turn, we conclude that the Pro704$^+$ allele is more common in Asian than Caucasian populations. This conclusion reveals an interesting link between a stronger suppressor allele and lower tumour incidence: Korean and Chinese have long been noted to have lower overall tumour incidence than Caucasians (Landis et al, 1999; Registry, 1995). Although it remains an unresolved issue whether genetic factors may exist to explain the disparity in tumour incidence between different racial groups, our data here suggests that gene polymorphisms can not be excluded.

### ACKNOWLEDGEMENTS

This work was support by NIH grant R01-CA76146 to SH. We thank Drs. A. Goldstein and P. Tucker for genomic DNAs of familial melanoma patients and C Polychronakos for genomic DNAs of normal individuals.

### REFERENCES

Abbondanza C, Medici N, Nigro V, Rossi V, Gallo L, Piluso G, Belsito A, Roscigno A, Bontempo P, Puca AA, Molinari AM, Moncharmont B, Puca GAB and Puca GA (2000) The retinoblastoma-interacting zinc-finger protein RIZ is a downstream effector of estrogen action. *Proc Natl Acad Sci USA* **97**: 3130–3135

Bale SJ, Dracopoli NC, Tucker MA, Clark WH, Jr, Fraser MC, Stanger BZ, Green P, Donis-Keller H, Housman DE and Greene MH (1989) Mapping the gene for hereditary cutaneous malignant melanoma-dysplastic nevus to chromosome 1p. *N Engl J Med* **320**: 1367–1372

Bardi G, Pandis N, Fenger C, Kronborg O, Bomme L and Heim S (1993) Deletion of 1p36 as a primary chromosomal aberration in intestinal tumorigenesis. *Cancer Res* **53**: 1895–1898

Baylin SB and Herman JG (2000) DNA hypermethylation in tumorigenesis: epigenetics joins genetics. *Trends Genet* **16**: 168–174

Buyse IM, Shao G and Huang S (1995) The retinoblastoma protein binds to RIZ, a zinc finger protein that shares an epitope with the adenovirus E1A protein. *Proc Natl Acad Sci USA* **92**: 4467–4471

Chadwick RB, Jiang G-L, Bennington GA, Yuan B, Johnson CK, Stevens MW, Niemann TH, Peltomaki P, Huang S and de la Chapelle A (2000) Candidate tumor suppressor RIZ is frequently involved in colorectal carcinogenesis. *Proc Natl Acad Sci USA* **97**: 2662–2667

Eng C, Herman JG and Baylin SB (2000) A bird's eye view of global methylation. *Nat Genet* **24**: 101–102

Fang W, Piao Z, Simon D, Sheu J-C and Huang S (2000) Mapping of a minimal deleted region in human hepatocellular carcinoma to 1p36.13–p36.23 and mutational analysis of the *RIZ (PRDM2)* gene localized to the region. *Genes Chromosomes Cancer* **28**: 269–275

Genuardi M, Tsihira H, Anderson DE and Saunders GF (1989) Distal deletion of chromosome Ip in ductal carcinoma of the breast. *Am J Hum Genet* **45**: 73–82

Hammond C, Jeffers L, Carr BI and Simon D (1999) Multiple genetic alterations, 4q28, a new suppressor region, and potential gender differences in human hepatocellular carcinoma. *Hepatology* **29**: 1479–1485

He L, Yu JX, Liu L, Buyse IM, Wang M-S, Yang Q-C, Nakagawara A, Brodeur GM, Shi YE and Huang S (1998) RIZ1, but not the alternative RIZ2 product of the same gene, is underexpressed in breast cancer, and forced RIZ1 expression causes $G_2$-M cell cycle arrest and/or apoptosis. *Cancer Res* **58**: 4238–4244

Huang S, Shao G and Liu L (1998) The PR domain of the Rb-binding zinc finger Protein RIZ1 is a protein binding interface and is related to the SET domain functioning in chromatin-mediated gene expression. *J Biol Chem* **273**: 15933–15940

Jiang G-L, Liu L, Buyse IM, Simon D and Huang S (1999). Decreased RIZ1 expression but not RIZ2 in hepatoma and suppression of hepatoma tumorigenicity by RIZ1. *Int J Cancer* **83**: 541–547

Jones PA and Laird PW (1999) Cancer epigenetics comes of age. *Nature Genetics* **21**: 163–167

Kuroki T, Fujiwara Y, Tsuchiya E, Nakamori S, Imaoka S, Kanematsu T and Nakamura Y (1995) Accumulation of genetic changes during development and progression of hepatocellular carcinoma: loss of heterozygosity of chromosome arm 1p occurs at an early stage of hepatocarcinogenesis. *Genes Chromosomes and Cancer* **13**: 163–7

Landis SH, Murray T, Bolden S and Wingo PA (1999) Cancer statistics, 1999. *Ca: a Cancer Journal for Clinicians* **49**: 8–31

Lin YW, Sheu JC, Huang GT, Lee HS, Chen CH, Wang JT, Lee PH and Lu FJ (1999) Chromosomal abnormality in hepatocellular carcinoma by comparative genomic hybridisation in Taiwan. *Int J Cancer* **35**: 652–658

Liu L, Shao G, Steele-Perkins G and Huang S (1997) The retinoblastoma interacting zinc finger gene RIZ produces a PR domain lacking product through an internal promoter. *J Biol Chem* **272**: 2984–2991

Muraosa Y, Takahashi K, Yoshizawa M and Shibahara S (1996) cDNA cloning of a novel protein containing two zinc-finger domains that may function as a transcription factor for the human heme-oxygenase-1 gene. *Eur J Biochem* **235**: 471–479

Piao Z, Park C, Park JH and Kim H (1998a) Allelotype analysis of hepatocellular carcinoma. *Int J Cancer* **75**: 29–33

Piao Z, Park C, Park JH and Kim H (1998b) Deletion mapping of chromosome 4q in hepatocellular carcinoma. *Int J Cancer* **79**: 356–60

Piao Z, Fang W, Malkhosyan S, Kim H, Horii A, Perucho M and Huang S (2000) Frequent frameshift mutations of RIZ in human gastrointestinal and endometrial carcinomas with microsatellite instability. *Cancer Res* **60**: 4701–4704

Rea S, Elsenhaber F, O'Carroll D, Strahl B, Zu-Wen S, Manfred S, Opravil S, Mechtler K, Ponting C, Allis C and Jenuwein T (2000) Regulation of chromatin structure by site-specific histone H3 methyltransferases. *Nature* **406**: 593–599

Registry CC (1995) *Cancer Incidence and Mortality in California by Detailed Race/Ethnicity, 1988–1992* (*www.ccrcal.org/calif95/index.htm*)

Sakurada K, Furukawa T, Kato Y, Kayama T, Huang S and Horii A (2001) RIZ, the retinoblastoma protein interacting zinc finger gene, is mutated in genetically unstable cancers of the pancreas, stomach, and colorectum. *Genes Chromosomes Cancer* **30**: 207–211

Shapiro VS, Lee P and Winoto A (1995) Identification and cloning of the G3B cDNA encoding a 3′ segment of a protein binding to GATA-3. *Gene* **163**: 329–330

Simon D, Knowles BB and Weith A (1991) Abnormalities of chromosome 1 and loss of heterozygosity on 1p in primary hepatomas. *Oncogene* **6**: 765–770

Weith A, Brodeur GM, Bruns GA, Matise TC, Mischke D, Nizetic D, Seldin MF, van Roy N and Vance J (1996) Report of the second international workshop on human chromosome 1 mapping 1995. *Cytogenetics and Cell Genetics* **72**: 114–144

*© 2001 Cancer Research Campaign*
